# An Observational Tool to Assess Activity Limitation in Ambulatory People with Cerebral Palsy When Performing Motor Skills

**DOI:** 10.3390/ijerph17061896

**Published:** 2020-03-14

**Authors:** Alba Roldan, José M. Sarabia, Guillermo Gómez-Marcos, Raul Reina

**Affiliations:** Sport Research Centre, Department of Sport Sciences, Miguel Hernández University, 03202 Elche, Spain; aroldan@umh.es (A.R.); jsarabia@umh.es (J.M.S.); guillegomezmarcos@hotmail.com (G.G.-M.)

**Keywords:** brain impairment, para-sport, adapted physical activity

## Abstract

Ratios of physical activity and sports participation in people with cerebral palsy (CP) are still low compared with people without a disability. For an adequate and useful practice, physical activity professionals should understand how different types of CP profiles constrain the performance of motor skills that are required during sports practice. This study aims to develop an observation-based assessment tool to evaluate activity limitations in individuals with a moderate level of CP when performing skills requiring jumping, sprinting, change of direction, coordination, and balance. Nineteen observers with different backgrounds from five world regions were recruited for this study, with accredited experience classifying/observing para-athletes with CP. All observers watched videos of 20 international para-athletes with different CP profiles (bilateral spasticity, athetosis/ataxia, unilateral spasticity; all Gross Motor Function Classification System level I) performing 16 motor tasks, and their observations were recorded throughout an ad-hoc data collection instrument. A total of 6080 units of qualitative information were recorded for data analysis. An observation-based tool with qualitative descriptors is derived from data analyses, describing how coordination and balance affected mainly in those with dyskinesia/ataxia, range of movement in those with diplegia, and asymmetries in those with hemiplegia. This tool would help sports practitioners and physical educators to better understand how different CP profiles constrain the performance of motor skills.

## 1. Introduction

Cerebral palsy (CP) is a group of permanent disorders affecting the development of movement and posture causing activity limitation, which is attributed to non-progressive disturbances that occurred in the developing fetal or infant brain [1]. The most common disorders are abnormal muscle tone (i.e., abnormal muscle stiffness), muscle weakness, random and uncontrolled body movements, and balance and coordination problems [2,3]. According to the Surveillance of Cerebral Palsy in Europe, CP can be classified in three major groups depending on type of motor impairment: spasticity, dyskinesia, or ataxia; and spastic profiles in two subgroups depending if one (i.e., unilateral) or both sides of the body are affected (i.e., bilateral) [4].

Lack of physical activity (PA) has been shown to be a serious concern for the whole population, but with special emphasis for people with disabilities who are at higher risk of suffering from health problems [5]. Even though people with disabilities are encouraged to practice more PA and sports, their participation rates are still low, particularly among the group with CP [6,7]. While active, performance-focused exercise with variable practice opportunities improves gross motor function in ambulant people with CP [8], developing healthy lifestyles when living with a disability can be a daunting task, as participation in PA requires minimum levels of strength, endurance, balance, and coordination [5]. In most cases, one or more of these physical attributes are impaired, resulting in a poor physical capacity that hinders tasks related to PA/sports practice, labor obligations, or household chores, constraining their involvement in community activities [9,10]. 

The International Classification of Functioning, Disability, and Health (ICF) model provided by the World Health Organization [11] indicates that the body functions (i.e., the physiological functions of body systems, including psychological functions) and/or body structures (i.e., anatomical parts of the body such as organs, limbs, and their components) are highly related to the activity limitation (i.e., difficulties an individual may have in executing activities). In people with CP, impaired body functions are usually coded as muscle tone functions (b735), control of voluntary movement functions (b760), involuntary movement functions (b7650), that are related with hypertonia, ataxia, and dyskinesia, respectively. Therefore, to support ambulatory individuals with CP when practice sports/PA, it is necessary to understand how an individual’s motor function limits the performance of specific motor tasks/skills [12] such as running [13,14], changing direction [15], jumping [16], accelerating/decelerating, or performing ball skills [17]. When sport specialists are able to identify the relationship between these two features (i.e., individual’s impairment—neuromusculoskeletal and movement-related functions—and the activity limitation that the CP profile has on motor skill execution—mobility), they are able to design appropriate tasks and adaptations or even making an accurate orientation on what type of sports are more feasible to practice depending on an individual’s profile [18]. This is especially important when a person with CP wants to practice sports at a competitive level, because in para-sport or sport for people with disabilities, there is what is called sports classification, which determines who is eligible to compete in one para-sport or another and determines in which sport class a person with a disability can compete, with the aim of creating a fair competition, regardless of the level of competition (e.g., local, regional, national, or international) [19]. 

Traditionally, in sports settings, the gross motor function of athletes with CP has been evaluated through the Cerebral Palsy International Sport and Recreation Association (CPISRA) classification system. The CPISRA system counts with eight classes of grouping athletes by similar capacities: the first four groups (classes 1–4) correspond to athletes who need a wheelchair to perform any sport/PA, while the last four groups (classes 5–8) host ambulant athletes. This classification has been used for exercise management in people with CP [20], and it has been applied for the last three decades in Paralympic sports such as boccia (classes 1 and 2), CP football (classes 5–8), or para-athletics (classes 1–8). In terms of clinical practice, the Gross Motor Function Classification System (GMFCS) [21] is a widely used tool, emerged from the Gross Motor Function Measure [22], to assess gross motor function in individuals with CP [23], and validated for use on individuals over 18 years of age [24,25]. The GMFCS is simpler than the CPISRA system, grouping individuals into five groups, from level I (i.e., independent movement) to level V (i.e., complete assistance is required). Although there are some provisions about the linkage of the GMFCS with the recommended sports practice by people with CP [18], nor CPISRA or GMFCS provide the linkage of the impairment with the activity limitation during the performance of motor skills or sports practice. 

The aim of this study is to develop an observation-based assessment tool to allow sports and PA practitioners to evaluate activity limitations in ambulatory para-athletes with a moderate level of CP when performing motor skills. In addition, the tool will provide qualitative descriptors by different subtypes of CP (i.e., diplegia or bilateral spasticity, athetosis/athetosis, hemiplegia or unilateral spasticity) identifying specific activity limitations when performing motor skills of running, jumping, changing of direction, coordination, and balance.

## 2. Materials and Methods 

### 2.1. Participants

This observational cross-sectional study was conducted with a sample of 19 international experienced classifiers (Table 1) with at least three years of international experience and five years of national experience in para-athletics and/or CP football classification. Three classifiers were a physician (15.8%), 11 physiotherapists (57.9%), and five sport technicians (26.3%). The sample of classifiers was representative of five regions: Africa (n = 3, 15.8%), Asia (n = 3, 15.8%), Europe (n = 4, 21%), Australasia (n = 5, 26.3%), and the Americas (n = 4, 21%). It was found that 64.3% of the classifiers had several years of experience training new classifiers, that is, showing skills to assess CP-related impairments and their linkage with the activity limitation to classify para-athletes with eligible impairments of spasticity, dyskinesia, or ataxia. 

In addition, out of a total number of 120 players who completed the battery of tests, a sample of 20 para-athletes (Table 2) was randomly selected for the aims of this study. All of them were classified as GMFCS Level I [21]. This group of para-athletes was composed of participants from the Americas (n = 14, 70%) and Europe (n = 6, 30%), all of them with international experience participating in world championships (n = 16, 80%) or Paralympic Games (n = 4, 20%) at least once.

All participants agreed to participate in the study and signed an informed consent form provided prior to participation. Ethical approval was obtained through the local University Ethics Committee (DPS.RRV.04.15).

### 2.2. Materials

Four international experts (i.e., three university professors with expertise in adapted PA and Paralympic sports, the heads of classification for CP football and para-athletics, and a full-time researcher in evidence-based classification for para-athletes) met to establish a consensus about the observation categories and the adequate test to assess each. They agreed on the following observation categories in which the impact of impairment would be observed during the tests, with wide applications to para-sports practiced by ambulatory people with CP: (i) coordination; as the ability to voluntarily execute fluid, accurate movements rapidly; (ii) balance; as the ability to maintain the line of gravity of the body within the base of support with minimal postural sway; (iii) range of movement (ROM); as the full movement or optimal potential of a joint, usually its range of flexion and extension; and (iv) symmetry; as the correspondence and/or movement similarity on opposite sides of a dividing line or plane. The test battery selected included tasks such as jumping, running, change of direction, coordination, and balance tests. Table 3 shows the names of the 16 tests according to their category, if that test was previously applied in people with CP, and if the original study reports test reliability. 

An ad-hoc data collection instrument (see Supplementary File S1) for these observational categories and tests was developed using Adobe Acrobat software (version Pro DC, Adobe Inc, San José, CA, USA). It was composed by a section for qualitative feedback with a list of specific activity limitation features (i.e., the qualitative checklist) followed by a blank box for a free description of other impaired features observed during the para-athletes´ performance for each test. Before the study, another three international senior classifiers from Africa, Europe, and Australasia regions reviewed the data collection tool. Minor edits were made after receiving feedback from them, and the observation tool was set at 100% agreement by the four international experts involved in its design.

Finally, during a session for classification purposes in summer 2013, the para-footballers’ performance in the 16 tests was video-recorded. The instructions given to the para-footballers and the full protocol of each test are detailed in Supplementary File S1.

### 2.3. Procedure

Twenty videos, one for each para-athlete, were edited using Windows Movie Maker (version 2013, Microsoft Corporation, Albuquerque, NM, USA) and were uploaded to a cloud server (Dropbox Inc, San Francisco, CA, USA). Every video contains all the 16 tests for one para-athlete and was randomly coded (A01 to A20) considering the para-athletes’ sport class to avoid any bias when observing individuals with the same CP profile: T35/FT5 (i.e., moderate spastic diplegia), T36/FT6 (i.e., moderate ataxia/athetosis), T37/FT7 (i.e., moderate spastic hemiplegia), or T38/FT8 (i.e., minimal impairment for athetosis, athetosis, or spasticity). Every video was linked to a single observation form, so every observer completed a total of 20 observations, requiring 60–90 min each. 

Classifiers completed the data collection form at the same time they were observing, through the videos, how the athletes were performing the tests (Instructions are in Supplementary File S1, pages 1–3). The observation process was individual and self-paced, and they had a period of three months for completing this task. Contact between observers was not allowed to ensure that responses were personal and not consensual. All the qualitative information was extracted from every single questionnaire: 19 classifiers × 20 para-athletes × 16 tests = 6080 units of information. Every unit of information was coded considering the decision made by the observer about the para-athletes´ sport class. 

### 2.4. Data Analysis

The qualitative data extraction was performed using Microsoft Office Excel software (version 2013, Microsoft Corporation, Albuquerque, NM, USA). Several rigor criteria were considered to evaluate the scientific quality of the research due to the qualitative nature of the information used for this study [32,33]: (i) credibility (i.e., confidence in the truth of the findings) was controlled conducting a triangulation process between experts before data collection; (ii) transferability, involving observers from the five world regions, with different background and experience in two para-sports; (iii) confirmability (i.e., the findings of the study are shaped by the respondents and not researcher bias in any way), guaranteeing the independence of the 10 observers during data collection and the researchers during data analyses; and (iv) dependability, which was enhanced by providing an in-depth methodological description to allow the study to be repeated.

With regards to the qualitative checklist, the number of potential observations (NPO) is calculated by the total amount of items per category x number of para-athletes x number of observers. On the other hand, the number of real observations (RO) is the percentage of the items clicked by the observers that they considered relevant for their decision-making. A 75% agreement across all the observers was necessary to include any item in the assigned category of coordination, balance, ROM, or asymmetry. On the other hand, two independent researchers analyzed the classifiers’ qualitative answers provided in the data collection forms into the categories and tests previously described. A minimum of 50% presence across observers were required to include that qualitative descriptor in the research outcome (i.e., the observation-based tool attached as Supplementary File S2). 

## 3. Results

Table 4 includes the percentage of agreements (i.e., checks/clicks) provided by the observers from the qualitative checklists, organized by the main performance features of coordination, balance, ROM, symmetry, and the type of impairment. The ROM received the highest percentage of clicks for those with lower limb spasticity (bilateral = 40%, unilateral = 48%); coordination was the category with the highest percentage of clicks for those with athetosis or ataxia (45%); and symmetry was highly scored for those with spasticity hemiplegia (39%). Balances were the observation category with the lowest scores for all the CP profiles. 

The qualitative information obtained from the blank boxes included for every test in the data collection form is provided, in a synthesized format, in Supplementary File S2 as an outcome for this research. That document includes the main activity limitation features that people with different CP profiles of diplegia, athetosis/ataxia, or hemiplegia exhibit when performing tests requiring jumping, sprinting, change of direction, coordination, or balance skills. Table 5 presents a few examples of qualitative feedback provided by classifiers for each observation category.

In addition to the four categories determined by the experts prior to data collection, extra information was obtained from classifiers on how impairments affect activity limitation when para-athletes were performing the observed motor skills. The researchers found this information so valuable that they added it into the final observation tool with the idea of helping sports technicians and physical educators to identify the relationship between impairment and activity limitation through practical examples.

## 4. Discussion

The literature has shown that sports technicians have difficulties in designing activities and experience difficulties when working with individuals with CP [34]. This study aimed to present a tool to understand how different CP profiles (i.e., bilateral spasticity, athetosis/ataxia, and unilateral spasticity) constraint the performance of several motor skills (i.e., activity limitation). The observational outputs were provided as a consensus among international classifiers with several backgrounds (i.e., medical doctor, physiotherapist, or sports technician) and with prominent experience with para-athletes with CP in physical activity and sports settings. 

The tool provided in this study would be relevant because the majority of available literature in sport/PA tends to consider all individuals with CP as a unique group/population, when each type of impairment has its own characteristics that should be considered when designing sports tasks. For example, regarding coordination, it can be seen that all CP profiles struggle when performing tasks requiring inter-limbs or gross coordination, but those with dyskinesia and ataxia face the biggest problems. Dyskinetic CP (both dystonia and athetosis) is characterized by involuntary, uncontrolled, recurring, and occasionally stereotyped movements, provoking abnormal patterns of posture and/or movement, especially on distal regions [35], while those with ataxia perform with low execution velocity that leads to poor running patterns [4]. Therefore, impaired coordination leads to a loss of orderly muscular sequencing, and movements are performed with abnormal force, rhythm, and inaccuracy, like jumping, as it is difficult to coordinate the limbs and core to perform an adequate push-off and/or landing [16,36].

Regarding balance, again, dyskinetic/ataxic profiles present greater problems, using compensatory strategies such as trunk sway or positioning hips forward and knee a little bit bent to keep balance. Translated to sport/PA tasks, those problems can be observed when performing dynamic activities requiring speed, like changes of direction, as they tend to lose balance when pivoting in one single leg; in starting, stopping, and turning when running [15]; or when they require object control (e.g., dribbling a ball in football), showing poor accuracy patterns [17]. 

Regarding the ROM, it should be mentioned that this is an intrinsic feature for individuals with hypertonia, due to the increased muscle tone provoking an increased resistance that is velocity-dependent [2]. Hypertonia is usually measured through the degrees of spasticity, so an increase in muscle tone would reduce the ROM of the body joints, especially with the presence of contractures [37] and muscles co-activation [38]. Those with this impairment usually present poor joint dissociation, especially in hips and ankles; abnormal body patterns due to stretch hyperreflexia; and compensatory strategies to keep balance, like an internal rotation of hips and ankles (i.e., scissor gait) and ankle clonus [35]. Thus, during sport/PA practice, it is common to find that individuals with spastic or mixed profiles present short stride lengths or short flight phases when running (poor knee elevation to keep balance) [17], poor push-off when jumping [16], or difficulties stopping in dynamic actions [39]. Consequently, impaired ROM by spasticity alters the skill biomechanics [36], like completing a full extension of the arm or leg after throwing or kicking actions. 

Finally, asymmetry limitations are also common in CP individuals with spastic profiles, especially in those with only one side of the body affected (i.e., unilateral spasticity or hemiplegia). Because many sports activities require inter-limbs coordination when performing (e.g., swimming, athletics, powerlifting, rowing, canoe, gymnastics), identifying the affected side and understanding that lack of symmetry is pertinent. These body asymmetries would be reflected as abnormal running patterns [40], difficulties manipulating objects with upper or lower limbs, loss of balance in jumping and landing phases [16], or difficulties when performing monopodal-support activities, especially with the most affected side [15,39].

Some limitations must be mentioned. First, coordination (e.g., b760), balance (e.g., b755), ROM (e.g., b710), and symmetry (e.g., b770) are categorized in the ICF as body function codes [11]. However, in this study, these dimensions have been used as qualitative descriptors to describe how impaired body function impacts on sports-related motor skills performance. Second, this study was conducted using observations on individuals with CP assessed as GMFCS Level I, the ablest profile. When individuals with disabilities present minimal impairments, identifying what impact the impairment has on activity limitation is more difficult. Therefore, the authors of this study are aware that the more severe the CP is, the easier it will be for sports technicians to identify the relationship between impairment and motor performance (i.e., activity limitation), but it should be also considered that active and performance-focused exercise would improve gross motor function in this population [8]. Three, this study involves only male para-athletes from a particular para-sport. Future studies should include female participants but also individuals from Africa or the Asia regions, as we did with the observers involved in this study.

## 5. Conclusions

The observation tool elaborated as an outcome from this research would help sports practitioners and physical educators to better understand how different CP profiles constrain the performance of motor skills. First, for those with bilateral spasticity or diplegia, performance is limited mainly because of muscles hypertonia in the lower extremities, usually in the legs, hips, and pelvis, constraining the ability for jumping (i.e., poor power output because of muscle co-contraction); changing direction, due to lower limb torsional deviations and pelvic malalignments; or sprinting (i.e., shorter strides). Second, those with dyskinesia and ataxia exhibit involuntary movements or impaired movement control, constraining the balance and fluency when performing motor skills. And three, for those with unilateral spasticity or hemiplegia, performance is limited mainly because of body asymmetries, also constraining their ability for jumping, with a lower involvement of the impaired leg at take-off and landing; changing direction (i.e., better performance and balance when pivoting on the less impaired side); or sprinting (i.e., asymmetrical strides and arms tandem). The qualitative information provided in the attached observation tool has recently been used to improve classification processes in para-sports such as CP football, providing descriptors of activity limitation for the three CP profiles used in this study [41]. This tool would help para-sports classifiers in decision-making, but it can also help the description of sport classes profiles of CP ambulant para-athletes (e.g., runners in Para athletics, classes T35-to-T38 [42]).

## Figures and Tables

**Table 1:** Characteristics of the international classifiers (i.e., observers).

	Physician	Physiotherapist	Sport Technician	Overall
Sex (M/F)	2/1	4/7	3/2	9/10
Age (yr)	47.3 ± 11.6	51.0 ± 11.0	41.7 ± 21.2	47.6 ± 11.3
Occupational Career (yr)	17.3 ± 14.0	28.0 ± 11.1	17.2 ± 17.0	22.7 ± 10.4
National Classifier (yr)	14.7 ± 13.6	17.4 ± 8.1	13.5 ± 16.3	15.7 ± 9.2
International Career (yr)	9.0 ± 2.5	8.1 ± 4.7	6.5 ± 4.2	7.9 ± 4.0

M = Male, F = Female, yr = Years, M ± SD.

**Table 2:** Characteristics of the para-athletes with cerebral palsy.

	Bilateral Spasticity or Diplegia	Dyskinesia or Ataxia	Unilateral Spasticity or Hemiplegia	Overall
N	4	7	9	20
Age (yr)	24.7 ± 4.7	24 ± 8.0	24.9 ± 5.7	24.5 ± 6.1
Height (cm)	177.8 ± 7.3	178.6 ± 8.0	170.8 ± 8.8	174.9 ± 8.7
Body weight (kg)	73.0 ± 4.0	68.5 ± 7.8	63.4 ± 7.0	67.3 ± 7.5
BMI (kg·m^−2^)	23.2 ± 2.5	21.3 ± 9.8	21.9 ± 1.2	22.0 ± 1.6
Experience (yr)	9.0 ± 5.3	9.6 ± 3.5	12.2 ± 8.4	10.6 ± 6.3

yr = years, cm = centimeters, kg = kilograms, BMI = body mass index, M ± SD.

**Table 3:** Summary of tests divided into categories.

Categories	Test	CP	Reliability (Type)	Reference
Jumping	Countermovement jump	Yes	Yes (intra)	Reina et al. (2018) [16]
	Four bounds for distance	Yes	Yes (intra)	Reina et al. (2018) [16]
	Standing broad jump	Yes	Yes (intra)	Reina et al. (2018) [16]
	Triple hop for distance	Yes	Yes (intra)	Reina et al. (2018) [16]
Running	10 m speed skip	No	Yes (inter)	Beckman and Tweedy (2009) [26]
	40-m sprint	Yes	Yes (intra)	Reina et al. (2017) [17]
	Stop and Go	Yes	Yes (intra)	Reina et al. (2017) [17]
CODA	Modified Agility Test	Yes	Yes (intra)	Reina et al. (2016) [15]
Coordination	Hexagon Agility Test	No	Yes (inter-s)	Beekhuizen et al. (2009) [27]
	Rapid heel-toe placement	No	No (n/a)	Bicici et al. (2012) [28]
	Running on place	No	Yes (inter-s)	Beckman and Tweedy (2009) [26]
	Side-stepping	No	Yes (inter-s)	Beckman and Tweedy (2009) [26]
	Split jumps	No	Yes (inter-s)	Beckman and Tweedy (2009) [26]
Balance	One leg stance	No	Yes (inter-r)	Springer et al. (2007) [29]
	Side-step	No	Yes (intra)	Fujisawa and Takeda (2006) [30]
	Tandem walk	Yes	Yes (intra)	Bar-Haim et al. (2013) [31]

CP = study applied in people with cerebral palsy, CODA = change of direction ability, intra = intra-session reliability, inter-s = between-sessions reliability, inter-r = inter-rater reliability, n/a = not available.

**Table 4:** The number of observations recorded from the qualitative checklist.

Title 1	Bilateral Spasticity or Diplegia		Dyskinesia or Ataxia		Unilateral Spasticity or Hemiplegia	
	NPO	RO (%)	NPO	RO (%)	NPO	RO (%)
Coordination	1830	30	2280	45	6030	28
Balance	1328	17	1588	28	4077	22
ROM	610	40	760	27	2010	48
Symmetry	976	28	1216	34	3216	39

NPO = number of potential observations; RO = real observations; ROM = range of movement.

**Table 5:** Examples of qualitative feedback provided by observers to describe activity limitation.

CP Profile	Category	Example of a Qualitative Description of Activity Limitation
Bilateral spasticity or diplegia	Coordination	“The athlete presents problems of coordination on distal joints in upper and/or lower limbs. A factor key to observe it is an appearance of clear coordination problems when fatigue appears.”
	Balance	“Athlete presents more problems in dynamic than static situations. The athlete uses compensatory strategies such as head movements to keep balance.”
	ROM	“Athlete presents high levels of spasticity which limits their ROM in both legs. The impact can be observed in a reduced extension of hip and knee elevation.”
	Symmetry	“The athlete presents moderate to severe asymmetry in both legs, but it might be present in upper limbs as well.”
Dyskinesia or ataxia	Coordination	“Athlete presents involuntary movements that impair coordination movement between upper and lower limbs, causing an awkwardness in movement quality. Due to these, athlete presents a high difficulty to dissociate movements, due to problems selecting hip-trunk muscles.”
	Balance	“Athlete has more challenge in backward actions (e.g. landing)”
	ROM	“Athlete uses compensatory strategies such as feet eversion.”
	Symmetry	“Athlete might present slight asymmetries between legs.”
Unilateral spasticity or hemiplegia	Coordination	“The athlete presents a difficulty to coordinate upper and/or lower limbs, causing an asymmetrical arm swing.”
	Balance	“Athlete uses compensatory strategies, such as abducting legs during landing or using a wider base.”
	ROM	“Athlete might present clearly limited ROM in the most affected side: reduced plantar and dorsal flexion in ankle joints. Due to poor dissociation, athletes might present very clear trunk rotation of hips to compensate and problems lifting feet and knee.”
	Symmetry	“Athlete presents clear and important asymmetry between both sides: upper and lower limbs.”

CP = cerebral palsy; ROM = range of movement.

## Supplementary Materials

The following are available online at https://www.mdpi.com/1660-4601/17/6/1896/s1, File S1: data collection form used main activity limitation features that people with different CP profiles of diplegia, athetosis/ataxia, or hemiplegia exhibit when performing the tests battery requiring jumping, sprinting, change of direction, coordination, or balance skills, File S2: main activity limitation features that people with different CP profiles of diplegia, athetosis/ataxia, or hemiplegia exhibit when performing the tests battery requiring jumping, sprinting, change of direction, coordination, or balance skills.
