# Supplementary material for: An Observational Tool to Assess Activity Limitation in Ambulatory People with Cerebral Palsy When Performing Motor Skills
Source: Int J Environ Res Public Health. 2020 Mar 14;17(6):1896. doi: 10.3390/ijerph17061896 (PMC7142872; doi:10.3390/ijerph17061896)
Supplement: Supplementary file 1 [file ijerph-17-01896-s001.zip › supplementary file S1.pdf]

supported by AGITOS Foundation

centro de investigación del deporte — Universidad Miguel Hernández de Elche

UNIVERSITAS Miguel Hernández

# Improving reliability and validity of current classification methods for athletes in classes T35 to T38

# Instructions and Data Collection Form

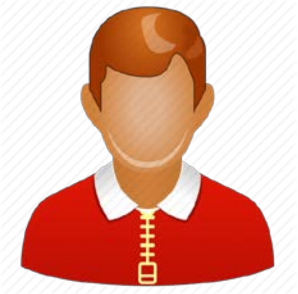

## Athlete ID:


| INDEX | PAGE |
| --- | --- |
| INSTRUCTIONS | 2 |
| HOW TO USE DATA COLL. FORM | 3 |
| DATA COLLECTION FORM | |
| 1. Side-Step test | 4 |
| 2. Rapid Heel-Toe Placement | 5 |
| 3. Split Jumps | 6 |
| 4. Side-Stepping | 7 |
| 5. Running in place | 8 |
| 6. Tandem Walk | 9 |
| 7. One Leg Stance | 10 |
| 8. Countermovement Jump | 11 |
| 9. Standing Broad Jump | 12 |
| 10. MAT Test | 13 |
| 11. Hexagon Hop Test | 14 |
| 12. Triple Hop for Distance | 15 |
| 13. Four Bounds for Distance | 16 |
| 14. 10m Speed Skip | 17 |
| 15. Stop and go | 18 |
| 16. 40 m Sprint | 19 |


VIDEO

PDF

# Instructions

**Before you start:**

1. Re-read the Participant Information Document, to remind yourself about:
   a. Your Role in the Project (i.e. what you need to do)
   b. the project materials
2. Read through all the instructions in this form and make sure you understand the process. Contact me if you have any questions.

**Materials:**

A reminder: data has been collected on 20 athletes. All are CP football players who competed at CPISRA ICUP 2013. Each athlete has been allocated an identification number from A01 to A20. Each athlete has a folder on dropbox which has their athlete ID on it (A01-A20) with two files in it – data collection form and a copy of the current class profiles. Videos are linked to the video icon in each data collection form (to can work on-line by video-streaming) or you can access through a Dropbox link (download and work off-line). The link to video files has been send by e-mail, together the link tou your personal Dropbox folder.

Open the folder for an athlete, open the two documents and the video (internet browser or video player), and arrange them so you can see all three at once. One way you could do this is to arrange them on your screen as the example below shows. However, if your screen is very small, this might not work, so an alternative would be to just have the video file and the data collection form open and print the class profiles and the definitions of the observation categories to manage it more easily. Another option would be to use 2 screens simultaneously (e.g., play the video through your TV, have the data collection form open in your PC and have printed copies of the class profiles).

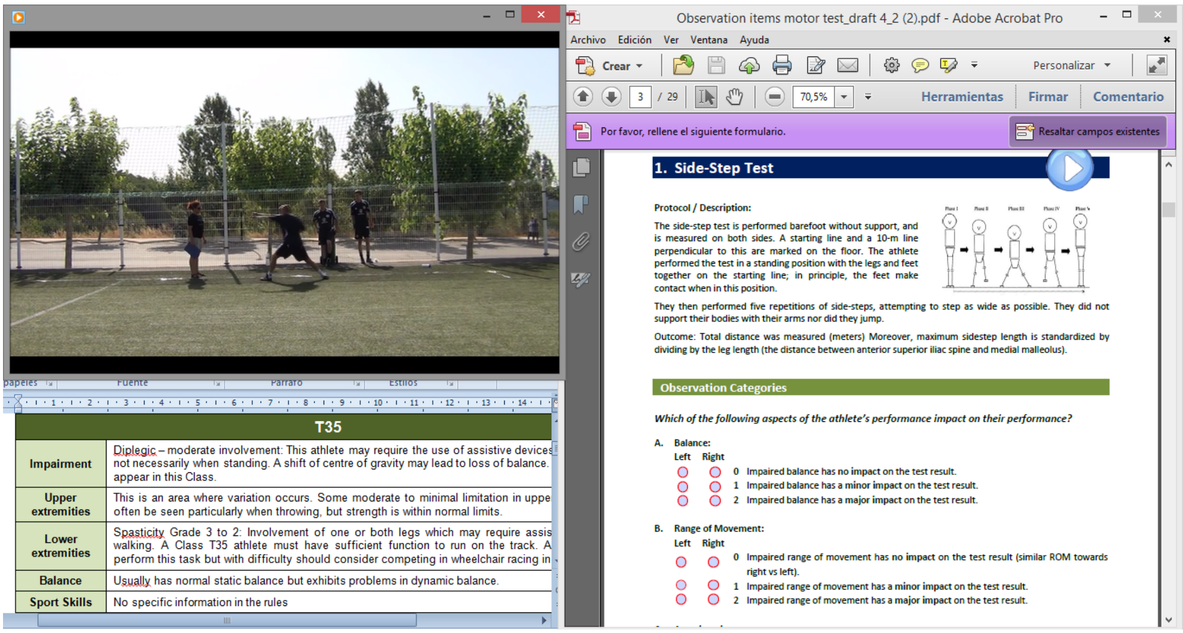


To complete this process:

- Play the first test on the video (sidestep test) and complete the questions on the data collection form for the side-step test.

# How to use the Data Collection Form?

Each Data Collection Form includes the following information about each test:

a) *Description of the test protocol.* You can get information about the instructions given to the athletes for each test.

b) Measured outcome. You can get information about the measured units used in each test.

c) *Picture or diagram*, showing each test.

*d) List of qualitative features of the performance*.

a. First, you can click **<u>one or several characteristics</u>** that you considered for your decision-making.

b. Next, in the *blank box*, you can write **<u>any additional features</u>** that you considered relevant for your decision-making or you think that have impact on athlete's performance. This information could help us in the future to impreve the class description.

# Data Collection Form

## 1. Side-Step Test

**Protocol / Description:**

The side-step test is performed barefoot without support, and is measured on both sides. A starting line and a 10-m line perpendicular to this are marked on the floor. The athlete performed the test in a standing position with the legs and feet together on the starting line; in principle, the feet make contact when in this position.

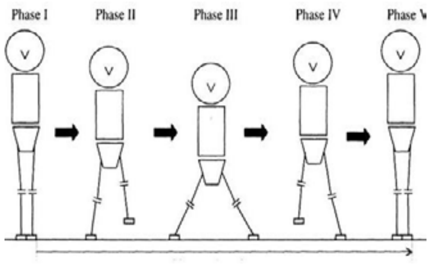


They then performed five repetitions of side-steps, attempting to step as wide as possible. They did not support their bodies with their arms nor did they jump.

Outcome: Total distance was measured (meters)

### Qualitative Feedback

*Describe the movement features that helped you decide your answer. For example:*

- ☐ The athlete loses fluency (among steps) during the movement.
- ☐ Lower limbs look different during the test; right vs. left (step length of foot position).
- ☐ Trunk stability varies among steps or has difficulty with trunk stability.

Did the athlete have any other movement characteristics which you thought had an impact on the test result?

| |
|---|
| |

# 2. Rapid Heel-Toe Placement

**Protocol / Description:**

The athlete sat barefoot on a chair and tried to touch the corners of 20 x 30 cm rectangle on floor. Athlete alternated heel and toe in each corner, first left to right (clockwise) then around right to left (anticlockwise). With the left foot, the athlete started from the bottom left corner, and with the right foot from bottom right corner.

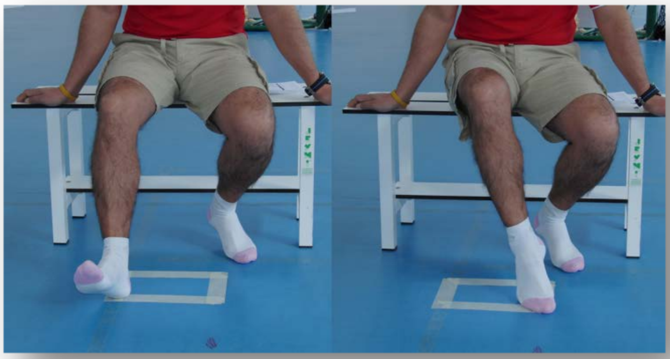

**Test Outcome:** Time (s) was measured for the fastest two trials, and number of incorrect contacts on corners was recorded.

Source: Proposed by an anonymous expert (2012) for Bicici, S., Tweedy, S. & Vanlandewijck, Y. (2012). Development of a test battery for improving classification reliability for ambulant athletes affected by hypertonia, ataxia or athetosis. KU Leuven: Unpublished Master Thesis.

## Qualitative Feedback

*Describe the movement features that helped you decide your answer. For example:*

☐ The athlete has to move from side to side a lot to accomplish the movement.

Did the athlete have any other movement characteristics which you thought had an impact on the test result?

| |
|---|
| |

# 3. Split Jumps

Athlete stood with legs slightly apart and one in front of the other. The athlete then jumped over a line by changing the leg position (Left in front, jump changing to Right in front). The arms were simultaneously moved contra-lateral to the legs.

**Outcome:** Time needed to complete 25 correct cycles, (seconds), and number of line touches.

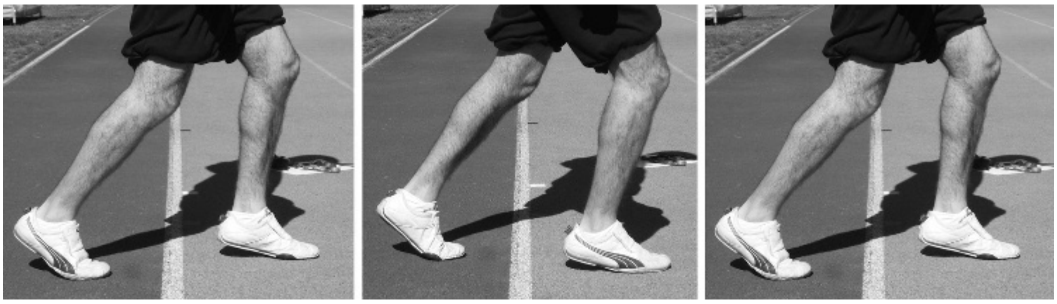

Source: Beckman, E.M. & Tweedy, S.M. (2009) Towards evidence-based classification in Paralympic athletics: evaluating the validity of activity limitation tests for use in classification of Paralympic running events. *British Journal of Sports Medicine*, 43, 1067-1072

## Qualitative Feedback

*Describe the movement features that helped you decide your answer. For example:*

- ☐ The athlete had difficulties in keeping the starting position.
- ☐ Requires some stops during the trial.
- ☐ Stepped on the line a lot.
- ☐ Arms did not move contralateral to legs.
- ☐ Flat steps or no contact with forefoot.
- ☐ Movement usually breaks down or athlete loses rhythm.

Did the athlete have any other movement characteristics which you thought had an impact on the test result?

# 4. Side Stepping

**Protocol / Description:**

Athlete stood with legs slightly apart between two lines separated at 40 cm then jumped over the line performing symmetrical abduction-abduction of the legs (open-close). The arms could be moved freely.

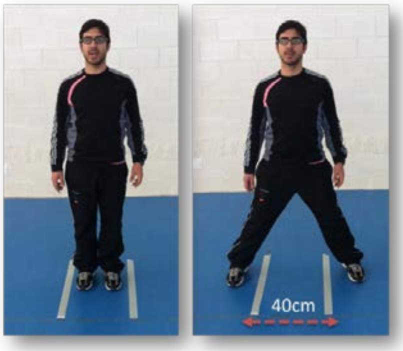


**Outcome:** Time needed to complete 15 correct cycles (seconds), and number of invalid trials (both feet did not step outside the line or inside the line)

## Qualitative Feedback

*Describe the movement features that helped you decide your answer. For example:*

- ☐ The athlete had difficulty in keep the starting position.
- ☐ Requires some stops during the trial.
- ☐ Stepped on the line a lot.
- ☐ Movement usually breaks down or athlete loses rhythm.
- ☐ Clear difference in movement right vs. left or vice versa.
- ☐ Feet steps flat (no forefoot).

Did the athlete have any other movement characteristics which you thought had an impact on the test result?

| |
|---|
| |

# 5. Running in Place

**Protocol / Description:**

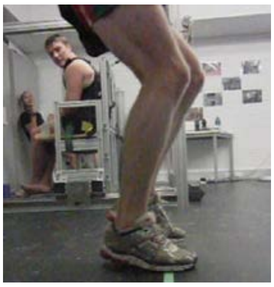

Participant stood with both feet next to each other. Participant ran on the same spot as fast as possible for 25 cycles. A cycle is right foot contact to next right foot contact. Tester said stop when 25 correct cycles were completed. Tester counted down: "Ready, Set, GO". Tester counted the correct cycles out loud, if there is an incorrect one, tester repeated the same number until the next correct cycle and counting upwards resumed.

**Outcome:** Time needed to complete 25 correct cycles (seconds)

Source: Beckman, E.M. & Tweedy, S.M. (2009) Towards evidence-based classification in Paralympic athletics: evaluating the validity of activity limitation tests for use in classification of Paralympic running events. *British Journal of Sports Medicine*, 43, 1067-1072

## Qualitative Feedback

*Describe the movement features that helped you decide your answer. For example:*

- The athlete had difficulty keeping the starting position or was stepping on the line:
  - ☐ Struggled to stay in the same spot on the floor during the whole test with large variations in position.
  - ☐ Athlete sometimes had difficulty staying in exactly the same spot but was able to maintain a similar position for the duration of the test.

☐ Required some stops during the trial.

☐ Flat steps or no contact with forefoot.

☐ Movement usually breaks down or athlete loses rhythm.

Did the athlete have any other movement characteristics which you thought had an impact on the test result?

# 6. Tandem Walk

**Protocol / Description:**

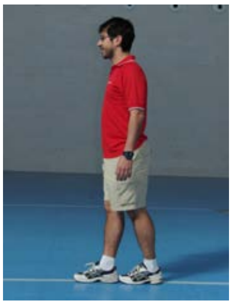

Participant walked barefoot, heel to toe, along a line as fast as possible and with best accuracy, with both arms crossed in front of chest. Two conditions were applied:

a) Time to complete 10 correct steps (heel needs to touch the toe during each step)
b) Time to complete 5m distance (heel-toe contact is not necessary)

**Outcome:** Time (seconds)

Source: Proposed by an anonymous expert (2012) for Bicici, S., Tweedy, S. & Vanlandewijck, Y. (2012). Development of a test battery for improving classification reliability for ambulant athletes affected by hypertonia, ataxia or athetosis. KU Leuven: Unpublished Master Thesis.

## Qualitative Feedback

*Describe the movement features that helped you decide your answer. For example:*

- ☐ Not able to contact along all the line / 10 steps.
- ☐ Contact during at least 50% of the test (2.5 m or 5 steps).
- ☐ Lose balance frequently / had difficulty following the line.
- ☐ Needed frequent steps out of the line to complete the test / wide steps out of the line.
- ☐ Sometimes lose balance, touching out of the line, but continued the test without noticeable stops.

Did the athlete have any other movement characteristics which you thought had an impact on the test result?

# 7. One Leg Stance

**Protocol / Description:**

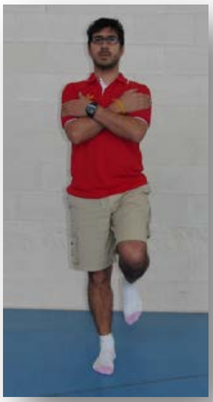

The player stood barefoot on a force platform. Prior to raising one leg off the floor, participants folded their arms across the chest. Stopwatch was started as soon as athlete lifted the foot off the floor. The player focused on a spot on the wall at eye level throughout the test.

**Outcome:** Mean score of the centre of pressure displacement (cm), measured on a force platform.

Source: Springer, B.A., Marin, R., Cyhan, T., Roberts, H. & Gill, N.W. (2007) Normative values for the Unipedal Stance Test with Eyes Open and Closed, *Journal of Geriatric Physical Therapy*, 30 (1), 8-15.

## Qualitative Feedback

*Describe the movement features that helped you decide your answer. For example:*

- Not able to perform the test with arms across chest:
  - ☐ The athlete moves the arms away from the body frequently.
  - ☐ Athlete sometimes moves the arms during the test.
- ☐ Moves the weight bearing foot frequently or sometimes.
- ☐ Not able to perform the test in an up-right position.
- ☐ Sometimes modifies posture (upright) to keep balance.

Did the athlete have any other movement characteristics which you thought had an impact on the test result?

# 8. Counter-Movement Jump

**Protocol / Description:**

Participants stood on a marked area (force platform) and, in their own time, jumped as high as they could, landing on both feet. Familiarization included standardized instructions, and participants placed their hands on the hips.

Three attempts were conducted and best score recorded.

**Outcome:** Height (cm)

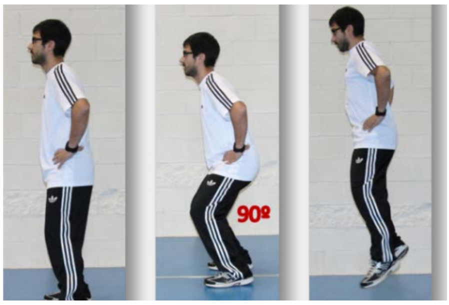


Source: Cámara, J., Grande, I., Mejuto, G., Los Arcos, A., & Yanci, J. (2013). Jump landing characteristics in elite soccer players with Cerebral palsy. *Biology of Sport*, 30(2)

## Qualitative Feedback

*Describe the movement features that helped you decide your answer. For example:*

- ☐ Poor coordination or balance may lead to trajectory that is not vertical.
- Landing:
  - ☐ Landing is non-stable and/or loose trunk/arms position.
  - ☐ Landing is stable but shows some loose of trunk/arms position.
- ☐ Athlete is heavily reliant on one leg or cannot use both effectively.

Did the athlete have any other movement characteristics which you thought had an impact on the test result?

# 9. Standing Broad Jump

**Protocol / Description:**

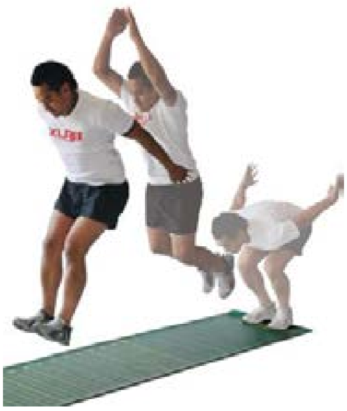

Participants stood on a line and, in their own time, jumped as far forward as they could, and landed on both feet. Familiarization included standardized instructions, and participants could use the stretch–shorten cycle and their arms to increase jump distance.

**Outcome:** Distance jumped (cm) is measured from the start line to the heel strike. Distance is divided by height for standardization.

Source: Beckman, E.M. & Tweedy, S.M. (2009) Towards evidence-based classification in Paralympic athletics: evaluating the validity of activity limitation tests for use in classification of Paralympic running events. *British Journal of Sports Medicine*, 43, 1067-1072

## Qualitative Feedback

*Describe the movement features that helped you decide your answer. For example:*

- ☐ Arms do not move forward symmetrically and powerful.
- ☐ Difference right <> left or noticeable incoordination /power during take-off.
- ☐ Difference right <> left or noticeable loss of balance during landing.
- ☐ Lack of coordination or fluency during flight phase.
- ☐ Landing is non-stable.
- ☐ Landing is stable but shows some posture instability.

Did the athlete have any other movement characteristics which you thought had an impact on the test result?

# 10. MAT Test

**Protocol / Description:**

Participant ran the MAT course as fast as possible with the following directions:

1) A-B movements (5 m): Participants sprinted forward to cone B and touch the top of it with the right hand.
2) B-C movements (2.5 m): Moving laterally without crossing the feet, participants ran to cone C and touched its top with the left hand.
3) C-D movements (5 m): Participants ran laterally to cone D and touched its top with the right hand.
4) D-B movements (2.5 m): Participants moved back to cone B and touched its top with the left hand.
5) B-A movements (5 m): Participants ran backwards to line A.

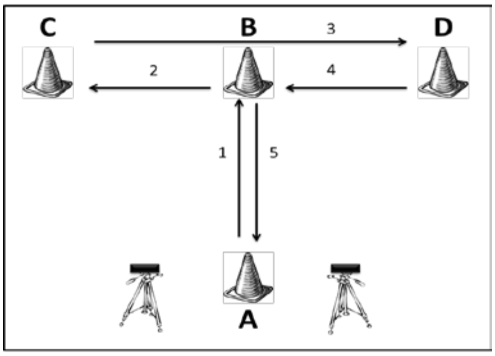


Trials where participants crossed their feet during B-C, C-D and D-B movements, failed to touch the top of the cone, and/or failed to face forward throughout the tasks, were repeated.

**Outcome:** time (s), measured with time gates.

Source: Yanci, J., Los Arcos, A., Reina, R., Gil, E., y Grande, I. (2014). La agilidad en alumnos de educación primaria: diferencias por edad y sexo. *Revista Internacional de Medicina y Ciencias de la Actividad Física y del Deporte*, *14* (53), 23-35.

## Qualitative Feedback

*Describe the movement features that helped you decide your answer. For example:*

- ☐ Noticeable activity limitation in running action forwards and backwards.
- ☐ Only shows activity limitation in backwards displacement (loose balance and uncoordinated movement).
- Lateral displacements (step):
  - ☐ One or two feet drag floor.
  - ☐ One or two feet drag slightly floor and is able to perform the movement after some coordination problems at the beginning.
- Lateral displacements (movement coordination):
  - ☐ Clear difference right <> left.
  - ☐ Slight difference right <> left: one leg push the other one.
- Speed at direction changes:
  - ☐ Clear asymmetric steps and difficulties to go down centre of gravity.
  - ☐ Requires speed deceleration to perform direction changes.
- ☐ Arms swing is poor or asymmetric, and no contributes to running speed.

Did the athlete have any other movement characteristics which you thought had an impact on the test result?

| |
|---|
| |

## 11. Hexagon Jump Test

**Protocol / Description:**

A hexagon with 60cm sides and 120-degree angles is marked with tape on a hard-surface floor with tape strip in the middle to mark the starting position. The test began with the subject standing on the tape strip placed in the middle of the hexagon, which marks the starting location. The tester gave the command ''Ready, go'' and starts the stopwatch. On the ''Go'' command, the participant began jumping (both legs) from the centre of the hexagon over each side and back to the centre in a clockwise direction until the participant went around the hexagon 3 times and returned to the centre (18 jumps). The stopwatch was stopped once the participant returned to the centre mark after 3 revolutions around the hexagon.

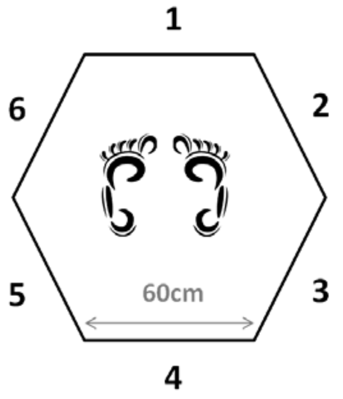


The participants faced the same direction during the course of the test, and the feet couldn't land on the taped edges of the hexagon or the trial was stopped and restarted. The participants were instructed to perform the test as fast as they could. No verbal inducements or encouragement were provided during the testing.

**Outcome:** Time to complete 3 revolutions (seconds)

Source: Beekhuizen, K.S., Davis, M.D., Kolber, M.J. & Cheng, M.S.S. (2009) Test-retest reliability and minimal detectable change of the hexagon agility Test. *Journal of Strength and Conditioning Research*, 23, 2167-2171.

## Qualitative Feedback

*Describe the movement features that helped you decide your answer. For example:*

- ☐ The athlete had difficulties to step on middle line before jumps.
- ☐ Requires some stops during the trial.
- ☐ Stepped out the hexagon side lines a lot.
- ☐ Movement usually breaks down or athlete loses rhythm.
- ☐ Difference right <> left and difficulties to still arms on hips.
- ☐ Touch the lines with feet but shows variability in his steps.

Did the athlete have any other movement characteristics which you thought had an impact on the test result?

## 12. Triple Hop for Distance

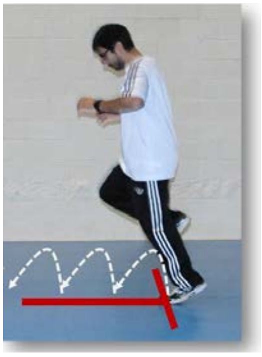

**Protocol / Description:**

Subjects were instructed to stand on one leg and perform 3 consecutive hops as far as possible, landing on the same leg.

Both limbs were tested (2 trials with each leg), and no restrictions were given to subjects regarding the use of arm movement (two valid trials with each leg)

**Outcome:** The total distance for 3 consecutive hops is recorded (m)

Munro, AG and Herrington, LC. (2011) Between-session reliability of four hop tests and the agility T test. *Journal of Strength and Conditioning Research*, 25(5), 1470-1477

### Qualitative Feedback

*Describe the movement features that helped you decide your answer. For example:*

- ☐ The athlete had difficulties to perform three consecutive hops (i.e requires some stops during the trial or loses balance during steps).
- ☐ Arms do not participate actively and contributes to hop.
- ☐ Feet dorsiflexion is weak during take-off and landing.
- ☐ Clear difference in performance left <> right.

Did the athlete have any other movement characteristics which you thought had an impact on the test result?

| |
|---|
| |

# 13. 4 Bounds for Distance

**Protocol / Description:**

Participants started on a marked line and were instructed to cover the maximum possible distance in four consecutive, single-leg bounds from a standing start. The first bound was from their non-preferred leg, landing on their outstretched preferred leg. Using forward momentum to continue the movement, the second bound was conducted as they leapt from their preferred leg to their non-preferred leg. This pattern was repeated for a total of 4 bounds. Distance is measured from the starting line to the heel strike of the fourth bound (m).

**Outcome:** Distance covered with 4 bounds (m)

Source: Beckman, E.M. & Tweedy, S.M. (2009) Towards evidence-based classification in Paralympic athletics: evaluating the validity of activity limitation tests for use in classification of Paralympic running events. *British Journal of Sports Medicine*, 43, 1067-1072

## Qualitative Feedback

*Describe the movement features that helped you decide your answer. For example:*

- ☐ The athlete had difficulties to bound (i.e. performs a step).
- ☐ Movement breaks down.
- ☐ Difference right <> left and difficulties to still arms on hips.
- ☐ Limited raising knee (i.e. leg doesn´t bend correctly).
- ☐ One knee bend more than the other one (i.e. close to hip line).
- ☐ The athlete does not use arms to push at the same time of the leg of each side.

Did the athlete have any other movement characteristics which you thought had an impact on the test result?

# 14. 10m Speed Skip

**Protocol / Description:**

Markers were placed at 0, 10 and 20 m with pairs of infrared timing light gates positioned at the 10 and 20 m markers. Participants performed the skip—a hop–step– hop pattern—and were given an opportunity to practice until they could successfully complete the pattern over 10 m. Participants accelerated over the first 10 m so that they were at top speed when they reach the first light gate (10 m), and maintained top-speed as they moved through to the second gate (20 m).

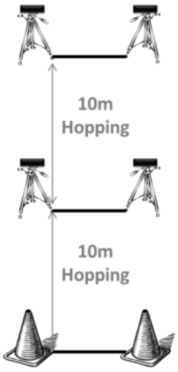


**Outcome:** Time (s) to move from 10 to 20 m was recorded.

Source: Beckman, E.M. & Tweedy, S.M. (2009) Towards evidence-based classification in Paralympic athletics: evaluating the validity of activity limitation tests for use in classification of Paralympic running events. *British Journal of Sports Medicine*, 43, 1067-1072

## Qualitative Feedback

*Describe the movement features that helped you decide your answer. For example:*

- ☐ Requires some stops during the trial.
- ☐ Movement usually breaks down or athlete loses rhythm.
- ☐ No clear flight phase or clear difference right <> left.
- ☐ Limited raising knee (i.e. leg doesn´t bend correctly).
- ☐ One knee bend more than the other one (i.e. close to hip line).
- ☐ The athlete does not use arms or arms push at the same time of the leg of each side.
- ☐ No active ankle dorsiflexion or difference right <> left.

Did the athlete have any other movement characteristics which you thought had an impact on the test result?

| |
|---|
| |

# 15. Stop and Go Test

**Protocol / Description:**

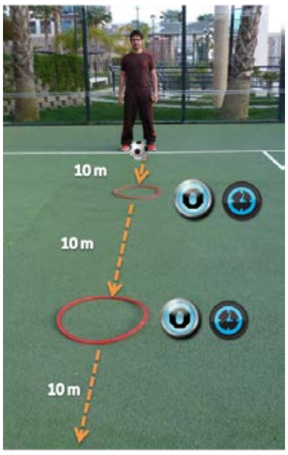


The athlete stood without support behind the starting line, and started to run at the researchers signal. The athlete ran to a mat (10m) and stopped completely on the mat with both feet. After the first contact, the athlete remained on the mat for 2 seconds until a beep sounded. Immediately at the sound they ran again to the next mat (10m) and stopped again until the next beep, and then continued to the final mark at 10m from the second mat. Total distance = 30m.

**Outcome:** time (seconds), measured with time gates to first mat (at 10m), second mat (at 20m), last gate (at 30m), total time (30m distance)

Source: Proposed by Reina, R. (2012) for Bicici, S., Tweedy, S. & Vanlandewijck, Y. (2012). Development of a test battery for improving classification reliability for ambulant athletes affected by hypertonia, ataxia or athetosis. KU Leuven: Unpublished Master Thesis.

## Qualitative Feedback

*Describe the movement features that helped you decide your answer. For example:*

- ☐ The athlete had difficulty stopping on the mats.
- ☐ The athlete uses always the same leg to start and stop.
- ☐ The athlete decreases speed significantly before reaching the mat.
- ☐ Flat steps or differences right <> left.
- ☐ The athlete does not use arms correctly for running (i.e. contralateral).

Did the athlete have any other movement characteristics which you thought had an impact on the test result?

# 16. 40m Sprint

**Protocol / Description:**

Athletes ran at maximum speed from a standing start to 40m. Timing light gates are positioned at 0, 10, 25 and 40 m

**Outcome:** time (s) to complete 10m, 25m and 40m.

Source: modified from Beckman, E.M. & Tweedy, S.M. (2009). Towards evidence-based classification in Paralympic athletics: evaluating the validity of activity limitation.

## Qualitative Feedback

*Describe the movement features that helped you decide your answer. For example:*

- ☐ The athlete does not use arms correctly for running (i.e. contralateral).
- ☐ No active legs push-off or noticeable differences right <> left.
- ☐ Weak active feet dorsiflexion or difference right <> left.
- ☐ The athletes uses trunk to speed up.
- ☐ Oscillation of centre of gravity or noticeable limp.

Did the athlete have any other movement characteristics which you thought had an impact on the test result?

| |
|---|
| |
